# Overview of the Drivers of Low-Value Care: Comment on "Key Factors that Promote Low-Value Care: Views of Experts From the United States, Canada, and the Netherlands"

**DOI:** 10.34172/ijhpm.2022.6833

**Published:** 2022-02-14

**Authors:** Joshua R. Zadro, Christopher G. Maher

**Affiliations:** ^1^Institute for Musculoskeletal Health, School of Public Health, Faculty of Medicine and Health, The University of Sydney, Sydney, NSW, Australia.; ^2^Sydney Local Health District, Sydney, NSW, Australia.

**Keywords:** Low-Value Care, Overuse, Underuse, Health Services, Healthcare

## Abstract

Verkerk and colleagues explored the key drivers of low-value care from the perspective of 18 policy-makers and researchers who had led and evaluated at least one initiative to reduce low-value care or had been responsible for reducing low-value care in an organisation. They identified several drivers of low-value care presented in the 2017 Lancet Right Care Series (eg, fee for service payment systems, the pharmaceutical and medical device industry, fear of malpractice litigation, issues with research conduct and reporting, a culture of ‘more is better’ and ‘new technology is better’) but did not discuss some other important ones. In this commentary, we aim to extend the work of Verkerk and colleagues and provide some additional perspectives on the drivers of low-value care within the following categories: *Economic incentives; Money, finance, and organisation; Knowledge beliefs, assumptions, bias and uncertainty; and Power and human relationships*.

 Verkerk and colleagues^1^ explored the key drivers of low-value care from the perspective of policy-makers and researchers who had experience in identifying and reducing low-value care. They conducted semi-structured interviews with a convenience sample of 18 participants from the United States, Canada, and the Netherlands who had led and evaluated at least one initiative to reduce low-value care (eg, Choosing Wisely) or had been responsible for reducing low-value care in an organisation. Key drivers of low-value care that emerged from the interviews were inter-related and categorised under system factors (fee for service payment systems, the pharmaceutical and medical device industry, fear of malpractice litigation), knowledge factors (issues with research conduct and reporting, medical education that is compromised by industry and encourages action rather than inaction) and social factors (a culture of ‘more is better’ and ‘new technology is better,’ patients requesting care based on misinformation). This study provides an important perspective on the drivers of low-value care based on the opinion of experts from three high-income countries. However, there would be value in considering how the opinions of the experts align with thinking in the field.

 The 2017 Lancet Right Care series provides the most comprehensive global overview of the evidence for overuse (a concept closely related to low-value care) and underuse,^2,3^ the drivers of poor medical care,^4^ and strategies to address overuse and underuse.^5^ The third article in the series – ‘Drivers of poor medical care’ – identified many important drivers of low-value care that were not offered by the participants in Verkerk and colleagues study.^1^ In this commentary, we aim to build upon the valuable insights offered by Verkerk and colleagues^1^ to provide additional perspectives on the drivers of low-value care. An important starting point for this discussion is acknowledging the complexity of defining low-value care and related concepts. Low-value care has been defined as care that, according to the best available evidence, provides little-to-no benefit to patients, is likely to cause more harm than benefit, and is too expensive given its benefits.^5^ While it may be appealing to use this succinct definition to categorise care as being either high- or low-value, determining the value or appropriateness of care is more complex. Evidence on benefits, harms and cost-effectiveness is often low-quality or not available, high-quality evidence is not always generalisable to patients, and patients’ values and preferences are sometimes misaligned with the best available evidence.^4^ In reality, the value of care occurs along a continuum from clearly appropriate for everyone to clearly inappropriate for everyone, with most care falling somewhere in the middle (‘grey zone’). Although we provide examples of ‘low-value care’ in this commentary, we acknowledge we are making this judgement based on population averages and there may be scenarios where some examples would be appropriate for a patient. A more detailed discussion on the complexity of defining and measuring low-value care (and related concepts) is beyond scope of this commentary but can be found in the first article in the Lancet Right Care Series.^2^ Below we summarise the key drivers of low-value care using the categories described in the Lancet Right Care Series^4^ to expand upon the drivers discussed by Verkerk and colleagues.^1^ We also outline possible solutions.

##  Economic Incentives as They Influence Clinician Behaviour, Hospital Behaviour, and Patient Behaviour

 The experts interviewed by Verkerk and colleagues^1^ identified payment structure (eg, fee-for-service) and the pharmaceutical and medical device industry as key system-related drivers of low-value care, but mostly focused on how these factors influence clinician behaviour. The Lancet Right Care series^4^ additionally discussed the influence of economic incentives on hospital and patient behaviour. Hospitals in many countries have switched from a ‘payment per day’ system – which encourages long lengths of stay and more inpatient care – to ‘payment per case’ (or per diagnosis) which is intended to encourage early discharge and reduce unnecessary care. However, ‘payment per case’ is not without its problems. If the price for a particular diagnosis is high relative to the costs of the associated admission and procedures, hospitals are incentivised to admit patients with this diagnosis. An example of this is in France, where switching to ‘payment per case’ increased use of potentially unnecessary cataract surgery and endoscopies.^6^

 Economic incentives also influence patient behaviour.^4^ Patients who pay for health insurance might be encouraged to use covered health services beyond what is needed because they feel they are entitled to do so. Co-payments – where the insurer provides a partial rebate for health services – are often implemented to discourage patients from seeking potentially unnecessary care, but this comes with its own issues. Co-payments may increase the likelihood of people underusing essential services (eg, decreased medication compliance) and reduce the demand for preventative services because people do not consider that the long-term benefits of these services are worth the cost. Use of co-payments is likely most appropriate for care that provides a small benefit but is not essential.

##  Money, Finance, and Organisation: Health Coverage, Resource Allocation and the Organisation of Care Delivery, Financing and Configuration of Health Systems, and Integration Across Levels of Care

 Money, finance, and organisation were major drivers of low-value care in the Lancet Right Care Series^4^ but were only briefly touched on by the participants in Verkerk and colleagues study.^1^ Inadequate health insurance coverage can prevent people from accessing essential care (eg, if people are uninsured or underinsured, or if a health insurer does not cover a specific service). However, coverage decisions do not always ensure only effective or cost-effective care is provided. A 2012 analysis of Australia’s Medicare Benefit Schedule (MBS) found that over 150 low-value health services were being funded,^7^ and a few years prior to this, approximately 5550 MBS funded services (97%) had not been formally evaluated for safety, effectiveness and cost-effectiveness (accounting for ~99% of MBS expenditure).^8^ A more rigorous process for adding interventions to coverage schedules could ensure ineffective and harmful care is not funded. For conditions where there are limited effective options, interventions with uncertain effectiveness could be funded for a specified time, with regular review as evidence emerges.

 In terms of health system configuration, the supply of health professionals or services in a region typically dictates use and can encourage unnecessary care.^4^ For example, more general practitioners or specialists in a region will increase visits to these health professionals, while less general practitioners in a region will increase the use of speciality services and hospital admissions and reduce the use of preventative services. Regulating the supply of services in an area is one option, but this is challenging once health systems are already established. Poor integration of care (fragmentation) is another important driver of low-value care as it encourages duplication of services (eg, imaging) and lack of preventive or palliative services.^4^

##  Knowledge Beliefs, Assumptions, Bias and Uncertainty: Flawed Production and Dissemination of Knowledge, Thinking Frameworks That Influence Decision-Making, Heuristics That Shape Thinking Frameworks, Common Assumptions of Modern Medical Culture, Dominance of the Biomedical Model, the Isolated Clinical Relationship 

 The participants in Verkerk and colleagues study^1^ identified that publication bias, the ambition of researchers, and industry-sponsored research can lead to research findings that overestimate the benefits of tests and treatments and mislead clinicians. The Lancet Right Care Series^4^ adds to this, highlighting the danger of underpowered studies, studies that use endpoints that are low priority, and research neglecting questions of functional, social and emotional wellbeing, adverse events and long-term outcomes. Solutions range from ensuring high-quality peer review of grants and journal articles to improving the ability of health professionals to appraise evidence. The Series also raises the important issue of flawed dissemination of evidence. New research often fails to reach those at the coalface and influence practice, while inaccurate online information misleads the public and encourages patients to request low-value care.^9,10^

 By largely focusing on national-level factors that promote low-value care, the participants in Verkerk and colleagues study^1^ missed some key drivers of low-value care related to knowledge, beliefs, assumptions, bias, and uncertainty.^4^ For example, patients often have unquestioning trust in a doctor’s expertise, the accuracy of a test or the effectiveness of a treatment, use anecdote to justify healthcare decisions (eg, “my friend had a good outcome with this treatment”), and are scared to ask their health professional questions. Patient decision aids can help patients better understand the benefits and harm of their options and be more active in healthcare decisions.^11,12^ Clinicians often disagree with clinical research that contradicts their practice or training and over-rely on pathophysiological and anatomical reasoning for providing a test or treatment. For example, some shoulder surgeons disagree with high-quality evidence that rotator cuff repairs provide limited clinical benefit for people with degenerative full-thickness rotator cuff tears, because they perceive a tear as something that needs to be repaired.^11^

 Heuristics (unconscious mental shortcuts) help health professionals make accurate clinical decisions based on a quick analysis of benefits and harms using diverse data and complex trade-offs. Yet, these mental shortcuts can create bias and increase use of low-value care.^4^ For example, an orthopaedic surgeon may interpret chest pain in a 60-year-old patient as musculoskeletal pain, while a cardiologist may interpret this as a heart problem (availability heuristic; the tendency to weigh likelihood of things by how easily they are recalled). Physicians might overestimate the benefit of cancer screening in young people as they are unaware of the low incidence of cancer in these groups (representative heuristic). A health professional might only search for and believe information that supports the benefits of treatments they use (confirmation bias).

 Quality of life and life expectancy have drastically improved over the last century, largely due to improved living conditions and public health intervention.^4^ However, people might incorrectly attribute these improvements to new medical interventions, increasing their expectations of the benefits of healthcare. People with this mindset might also adopt a biomedical model of thinking where any deviation from the biological norm (eg, imaging finding, blood test result) leads them to pursue further tests and treatment that may not be necessary.

##  Power and Human Relationships: Strengths or Weaknesses of the Therapeutic Relationship, Flawed Decision-Making, Contest for Political Control, Political Mobilisation and Demand for Care, Professional Societies and Other Mediators, and Fear of Litigation

 Fear of litigation was discussed at length in the article by Verkerk and colleagues.^1^ Yet, there are other factors related to power and human relationships that can exert their force on the provision of low-value care. The patient-clinician relationship at the point of care is ultimately where low-value care manifests. Imbalances of power can prevent shared decision making and lead to patients feeling pressure to take their health professionals advice without asking questions. Health professionals often do not have enough time to engage in shared decision making or convey complex information in an easy-to-understand way, and act according to what they consider to be in their patient’s best interest.^4^ However, trials of shared decision making suggest around 20% of elective surgeries would be unwanted if patients were appropriately informed of the benefits and harms and were not under time pressure to make decisions.^12^ Patients and health professionals might have different values and preferences, that without shared decision making would not be explored. A young health professional might have a different assessment of risk and values to an older patient. A wealthier health professional may not understand the economic concerns or trade-offs of a less wealthy patient. High-quality patient decision aids could help health professionals convey complex information in an easy-to-understand way, give patients an opportunity to reflect on their options, and engage patients in shared decision making.

 Another driver of low-value care is the contest for political control, political mobilisation, and demand for care.^4^ Commercial entities label new technologies as innovative and lifesaving, often distracting from the fact these new technologies are untested. These are then promoted by the media who are in pursuit of expanding the audience for their sponsors. Professional societies value prestige, respect, and market share within the community, and can sometimes resort to partisan tactics (either intentionally or unintentionally) to make their profession seem more evidence-based than others. For example, an analysis of Choosing Wisely recommendations found that many professional societies only campaign against low-value care if the care does not generate income for their members.^13^ Most people would consider that private insurance companies have an interest in reducing costs in the short term by reducing low-value care. However, private health insurers generate more revenue with increasing healthcare costs because it is a percentage of their total premium, making it less appealing for them to reduce low-value care.

 Demand for care can be driven by industry and professional associations.^4^ Direct-to-consumer advertising encourages consumers to request drugs or medical products through increased awareness of the “benefits” of drugs and harms of a disease (eg, sildenafil for erectile dysfunction, finasteride for baldness, enoxaparin for blood clotting, dabigatran etexilate for atrial fibrillation). Outlawing direct-to-consumer advertising is a clear solution adopted by many countries, but there are other ways to increase demand for care. Disease-awareness campaigns are typically alliances between industry and consumer groups that aim to increase fear of disease (eg, social anxiety disorder, restless leg syndrome, female sexual dysfunction) and increase the sale of medical products. Professional societies can also be responsible for expanding disease definitions and defining treatment thresholds. Hypertension is a key historical example of medical labels and treatments expanding from the sick to healthy. In 2003, the label “pre-hypertension” was controversially created by a group of experts – 82% of whom had ties to a median of 12 drug companies each.^14^ In 2017, the US Heart Association guidelines lowered the threshold further, turning “prehypertension” into “hypertension” (130+) – dramatically expanding the label to almost 46% of US adults and encouraging more people to seek drugs treatments.^15^

 We thank Verkerk and colleagues1 for initiating a discussion on drivers of low value care drawing from the perspectives of 18 experts in the field. We have searched the literature to provide additional perspectives on the drivers of low value care and possible solutions that we hope complements Verkerk and colleagues’ important paper. What is outside the scope of both our papers is a systematic review of the evidence on interventions that aim to replace low value care with the right care. We hope our commentary might encourage a review on this topic.

## Ethical issues

 Not applicable.

## Competing interests

 Authors declare that they have no competing interests.

## Authors’ contributions

 JRZ: conception and design, drafting and revision of the manuscript, and final approval of the version to be published. CGM: conception and design, drafting and revision of the manuscript and final approval of the version to be published.
